# Characterization of three rapidly growing novel *Mycobacterium* species with significant polycyclic aromatic hydrocarbon bioremediation potential

**DOI:** 10.3389/fmicb.2023.1225746

**Published:** 2023-09-08

**Authors:** Yang Deng, Tong Mou, Junhuan Wang, Jing Su, Yanchun Yan, Yu-Qin Zhang

**Affiliations:** ^1^Institute of Medicinal Biotechnology, Chinese Academy of Medical Sciences and Peking Union Medical College, Beijing, China; ^2^State Key Laboratory of Dao-di Herbs, Beijing, China; ^3^Graduate School of Chinese Academy of Agricultural Sciences, Beijing, China

**Keywords:** *Mycobacterium adipatum*, *Mycobacterium deserti*, *Mycobacterium hippophais*, genome, bioremediation potential

## Abstract

*Mycobacterium* species exhibit high bioremediation potential for the degradation of polycyclic aromatic hydrocarbons (PAHs) that are significant environmental pollutants. In this study, three Gram-positive, rapidly growing strains (YC-RL4^T^, MB418^T^, and HX176^T^) were isolated from petroleum-contaminated soils and were classified as *Mycobacterium* within the family *Mycobacteriaceae*. Genomic average nucleotide identity (ANI; < 95%) and digital DNA–DNA hybridization (dDDH; < 70%) values relative to other *Mycobacterium* spp. indicated that the strains represented novel species. The morphological, physiological, and chemotaxonomic characteristics of the isolates also supported their affiliation with *Mycobacterium* and their delineation as novel species. The strains were identified as *Mycobacterium adipatum* sp. nov. (type strain YC-RL4^T^ = CPCC 205684^T^ = CGMCC 1.62027^T^), *Mycobacterium deserti* sp. nov. (type strain MB418^T^ = CPCC 205710^T^ = KCTC 49782^T^), and *Mycobacterium hippophais* sp. nov. (type strain HX176^T^ = CPCC 205372^T^ = KCTC 49413^T^). Genes encoding enzymes involved in PAH degradation and metal resistance were present in the genomes of all three strains. Specifically, genes encoding alpha subunits of aromatic ring-hydroxylating dioxygenases were encoded by the genomes. The genes were also identified as core genes in a pangenomic analysis of the three strains along with 70 phylogenetically related mycobacterial strains that were previously classified as *Mycolicibacterium*. Notably, strain YC-RL4^T^ could not only utilize phthalates as their sole carbon source for growth, but also convert di-(2-ethylhexyl) phthalate into phthalic acid. These results indicated that strains YC-RL4^T^, MB418^T^, and HX176^T^ were important resources with significant bioremediation potential in soils contaminated by PAHs and heavy metals.

## Highlights

- Polycyclic aromatic hydrocarbons (PAHs) are significant environmental pollutants caused by human industrial activities.
- The bioremediation of PAHs is a promising method of pollutant mitigation and *Mycobacterium* species have been previously reported to hold considerable bioremediation potential.
- In this study, three novel species of *Mycobacterium* were isolated from polluted soil samples, formally characterized, and evaluated for bioremediation potential.
- The three strains were found to exhibit considerable potential for bioremediation of PAHs due to their physiological and genomic characteristics.
- Thus, these newly described species represent an important resource for bioremediation efforts.

## Introduction

The *Mycobacterium* genus encompasses a large group of Gram-positive, rod-shaped, acid-fast bacteria of the phylum *Actinomycetota*, that was first proposed by Skerman et al. (1980). Early classification of *Mycobacterium* was based on growth rate, pigmentation, and clinical significance (Runyon, 1959). Indeed, the fundamental taxonomic division of the group was connected to growth rate, such that species were defined as either slow or rapid growers. Rapid growers exhibit visible growth from dilute inocula within 7 days, while slow growers require over 7 days to achieve visible growth. Subsequently, immunological methods, in addition to comparisons of cell wall components, homologous enzyme sequences, DNA–DNA homologies, plasmid profiles, and restriction endonuclease analyses were used to complement the quantitative growth studies and infer natural relationships among *Mycobacterium* strains. Stahl and Urbance conducted a thorough phylogenetic analysis of *Mycobacterium* in 1990 based on comparison of 16S rRNA sequences, revealing phylogenetic relationships that were consistent with previous classifications (Stahl and Urbance, 1990). Lévy-Frébault and Portaels further proposed minimal standards for describing *Mycobacterium* species in 1992 (Lévy-Frébault and Portaels, 1992). These standards included acid-alcohol staining fastness, DNA G + C content levels, and mycolic acid presence. The recommended minimal standards for describing a new slowly growing *Mycobacterium* species were consequently based on phenotypic and genomic analyses. Takewaki et al. (1994) revealed species-specific restriction site profiles within amplified *dnaJ* genes in 1994 that could differentiate most *Mycobacterium* species based on a combination of PCR and restriction fragment length polymorphism analyses. The relationships among mycobacterial species were also concomitantly evaluated using the sequences of 23S rRNA gene spacers (Stone et al., 1995) in addition to those of several housekeeping genes including *hsp65* (Kim et al., 2005), *gyrB* (Kasai et al., 2000), *rpoB* (Tortoli, 2012), and *gyrA* (Guillemin et al., 1995). Gupta et al. (2018) proposed a revision of mycobacteria taxonomy in 2018 that redistributed the 150 species of *Mycobacterium* into five genera based on synapomorphies. The four newly proposed genera comprised the non-tuberculous mycobacteria and included *Mycobacteroides*, *Mycolicibacter*, *Mycolicibacterium*, and *Mycolicibacillus*. Non-tuberculous mycobacteria exhibit diverse genomic backgrounds and physiological characteristics, but produce remarkably similar disease manifestations within at-risk populations. Consequently, Tortoli et al. (2019) suggested not renaming clinically important organisms in 2019, including non-tuberculous mycobacteria, suggesting that the use of the previously established *Mycobacterium* genus exhibited the advantage of avoiding confusion in health care settings. The genus *Mycobacterium* currently comprises 195 species with validly published names.^1^ The primary ecological niche for some *Mycobacterium* is the diseased tissue of warm-blooded hosts. Most human infections are caused by either *M. leprae* or *M. tuberculosis*, although other *Mycobacterium* strains are opportunistic pathogens of humans, particularly in immunocompromised individuals (Hartmans et al., 2006). However, most *Mycobacterium* species are non-pathogenic to humans and are inhabitants of many natural environments, including freshwaters and soils (Falkinham, 2009). In addition, several strains of *Mycobacterium* have been isolated from soils contaminated with polycyclic aromatic hydrocarbons (PAHs) like gasoline and coal tar (Willumsen et al., 2001; Hormisch et al., 2004; Zhang et al., 2012).

PAHs have become a significant environmental concern due to their potential toxic, mutagenic, and carcinogenic properties (Zeng et al., 2004). *Mycobacterium* species are typical PAH-degrading bacteria and can degrade high molecular weight PAHs by introducing two oxygen atoms to PAH compounds through dioxygenase enzymes (Khan et al., 2001). *M. vanbaalenii* PYR-1 was first shown to degrade low molecular weight compounds like 4-ring pyrene (Pereira et al., 2020). The unique cell wall layer of mycobacteria, including the presence of mycolic acids, plays an important role in mycobacterial degradation of hydrophobic PAHs (Abbasnezhad et al., 2011). Indeed, pyrene-degrading mycobacteria can degrade diverse PAHs, including naphthalene, phenanthrene, and fluoranthene, and even five-ring compounds like benzo[α]pyrene via co-metabolic biodegradation, subsequently using these compounds as carbon sources (Kweon et al., 2011). Zeng et al. (2017) revealed that the PdoAB dioxygenase from *Mycobacterium* sp. NJS-P exerts versatile functions in oxidizing various PAHs, including up to five-ring compounds like benzo[α]pyrene. Further, *pdoAB* can be induced during bacterial growth on pyrene and phenanthrene, implicating its function in PAH degradation. *Mycobacterium* species in rhizosphere soils have also been shown to accelerate the degradation of organic contaminants like PAHs and enhance plant resistance to soil pollution (Dai et al., 2020). Moreover, Yang et al. (2021) recently observed that the pyrene-degrading bacterium, *Mycobacterium* sp. Pyr9 exhibited diverse plant growth-promoting characteristics and a high tolerance to harsh environments, suggesting a high potential for bioremediation application (Yang et al., 2021). Thus, pyrene-degrading *Mycobacterium* strains may contribute to improving the safety of agricultural products and human health in PAHs polluted environments.

Phthalates are widely used industrial compounds and intensively studied environmental pollutants that exhibit endocrine disrupting properties. Strain NK0301 was the first *Mycobacterium* strain observed to degrade phthalates. It could degrade di-(2-ethylhexyl) phthalate (DEHP) into 2-ethylhexanol and 1,2-benzenedicarboxylic acid (Nakamiya et al., 2005). Wright et al. (2020) subsequently observed that the marine phthalate-degrading strain *Mycobacterium* sp. DBP42 could degrade diverse phthalates by transforming them into phthalic acid (PA) via di-alkyl phthalate esters (DAPs), and was also able to utilize PA as a growth substrate via the protocatechuate branch of the *β*-ketoadipate pathway.

Notably, some rapidly growing mycobacterial strains that were previously classified as *Mycolicibacterium* are also phthalate-degrading bacteria. Fourteen molecular markers (4 CSIs and 10 CSPs) are unique to members of the former *Mycolicibacterium* genus, supporting its monophyly and genetic cohesiveness. These strains could be distinguished from other *Mycobacteriaceae* genera, in addition to other bacteria, based on the presence of conserved signature indels in genes encoding the LacI family transcriptional regulator, cyclase, CDP-diacylglycerol–glycerol-3-phosphate 3-phosphatidyltransferase, and CDP-diacylglycerol–serine O-phosphatidyltransferase (Gupta et al., 2018). A novel DEHP-degrading marine bacterium was identified in 2021 as *Mycolicibacterium phocaicum* RL-HY01 that was isolated from intertidal sediments polluted by municipal wastewaters. The strain could transform DEHP into PA by *β*-oxidation and de-esterification and was further utilized within the gentisate branch of the *β*-ketoadipate pathway (Ren et al., 2021). Strain YC-RL4^T^ was previously isolated from petroleum-contaminated soil and belonged to the *Mycobacterium* genus. The strain could utilize phthalates as their sole carbon source for growth and could also transform DEHP into PA via MEHP (mono (2-ethylhexyl) phthalate), with PA further being utilized for growth via the benzoic acid (BA) degradation pathway (Ren et al., 2016). Currently, limited research could be referred on the degradation of polycyclic aromatic hydrocarbons (PAHs) in environmental pollution by strains of the genus *Mycobacterium*. Especially, the taxonomic status of these PAHs-degrading strains and the clear understanding of the functional genes and metabolic pathways involved in PAHs degradation are still unclear. In this study, strain YC-RL4^T^ (= CPCC 205684^T^ = CGMCC 1.62027^T^) and two other strains that were closely related to each other were characterized, and their functional genes, metabolic pathways and potential for degrading PAHs were evaluated. Furthermore, this study also identified that these strains with PAHs degradation potential also possessed the potential heavy metal resistance. This remarkable bioremediation potential held significant importance for their future applications in the field of bioremediation. Polyphasic taxonomic analyses revealed that the three strains were novel *Mycobacterium* species with considerable application potential for the bioremediation of PAH-degrading soils.

## Materials and methods

### Strain acquisition

Strain YC-RL4^T^ was isolated from a petroleum-contaminated soil sample collected from Heze City (35°16′ N, 115°28′ E), Shandong Province, China, as described by Ren et al. (2016). Strain MB418^T^ was isolated from a gravel soil sample collected from the Gurbantunggut desert (45°22′ N, 88°20′ E) in Xinjiang. Strain HX176^T^ was isolated from rhizosphere soil associated with a medicinal plant in Xinjiang (43°36′ N, 82°11′ E). About 2 g of each sample was suspended in 18 mL of 0.85% (w/v) NaCl solution. Then, 200 μL of 10^−4^ diluted soil suspensions were spread on humic acid agar medium, as previously described (Deng et al., 2022). After incubation for 2 weeks at 28°C, visible colonies were picked and streaked on peptone yeast glucose (PYG) medium containing peptone (3 g L^−1^), yeast extract (5 g L^−1^), glycerol (10 g L^−1^), betaine hydrochloride (1.25 g L^−1^), sodium pyruvate (1.25 g L^−1^), and agar (15 g L^−1^), with adjustment to pH 7.2. The cultures were again incubated at 28°C to obtain isolated colonies. Purified isolates were maintained in glycerol suspensions (20%, v/v) at −80°C.

The reference strain *M. fluoranthenivorans* JCM 14741^T^ was obtained from the Japan Collection of Microorganisms (JCM), while strains *M. frederiksbergense* DSM 44346^T^ and *M. litorale* DSM 45785^T^ were acquired from the German Collection of Microorganisms and Cell Cultures (DSMZ).

### Phenotypic properties

Strain growth was evaluated on glucose–yeast extract–malt extract agar (GYM; DSMZ medium 65), Middlebrook 7H10 agar (MB7H10; Difco), nutrient agar (NA; Difco), and tryptic soy agar (TSA; Difco), with incubation at 28°C for 48–72 h. Cell motility was examined by microscopic observations and inoculation on semisolid GYM medium with 0.3% agar (w/v). The growth conditions of strains YC-RL4^T^, MB418^T^, and HX176^T^ were evaluated over temperature, pH, and salt tolerance ranges. The temperature range of growth was determined in MB7H10 mediu (Difco) at 4, 15, 20, 22, 25, 28, 30, 32, 35, 37, 45, and 50°C. The growth range across pH was determined in GYM medium at pH 4.0–10.0, at intervals of 1.0 unit. NaCl tolerance was determined with cultivation at 28°C in modified GYM medium (pH 7.0, without Na^+^ and Cl^−^), and with NaCl supplemented at concentrations of 0–10.0% (w/v) at increasing increments of 1.0%. All growth condition assays were performed in triplicate.

Biochemical and physiological tests were conducted for strains YC-RL4^T^, MB418^T^, HX176^T^ and other closely related strains. Oxidase activity was investigated using the API oxidase reagent (bioMérieux) according to the manufacturer’s instructions. Catalase activity was evaluated via production of bubbles after addition of a drop of 3% (v/v) hydrogen peroxide. Voges–Proskauer, H_2_S production, and starch hydrolysis tests were evaluated using a biochemical identification kit (Huankai Microbial) according to the manufacturers’ instructions. Additional biochemical characteristics including enzymatic activities, acid production from fermentation, and assimilation of carbon sources were evaluated using API ZYM, API 50CH, and Biolog GEN III MicroPlates (Biolog) according to the manufacturers’ instructions. API strip and Biolog results were recorded every 24 h after incubation at 28°C until all reactions had stabilized. The API and Biolog tests were performed in duplicate using consistent conditions.

### Chemotaxonomic characterization

Biomass for chemotaxonomic investigations of strains was obtained by cultivation in flasks on a rotary shaker (180 r.p.m.) using ISP2 broth as the medium and with incubation at 28°C for 3 days, except that cellular fatty acids and cell-wall mycolic acids extraction and analyses were conducted using cultures grown on Middlebrook 7H10 (MB7H10) medium. Cellular polar lipids were extracted, detected with two-dimensional TLC silica-gel 60 F_254_ thin-layer plates (10 × 10 cm, Merck), and analyzed, as previously described (Minnikin et al., 1984). Menaquinones were extracted and purified using reverse-phase HPLC, also as previously described (Minnikin et al., 1984). Cellular fatty acids were extracted, methylated, and analyzed using the Sherlock Microbial Identification System (MIDI) with the standard ACTIN1 database (version 6.0) according to the manufacturer’s instructions (Sasser, 2006). Diagnostic isomers of diaminopimelic acid in whole cell hydrolysates (6 N HCl, 120°C, 30 min) of the strains were subjected to thin-layer chromatography on cellulose plates (10 × 20 cm, Merck) using a previously described solvent system (Schleifer and Kandler, 1972). The cell-wall mycolic acids were determined following saponification, extraction and derivatization, and then separated using a gradient of methaand 2-propanol via high-performance liquid chromatography (HPLC) as recommended by the Sherlock Mycobacteria Identification System Operating Manual Version 1.0 (MIDI) (Butler and Guthertz, 2001; Zimenkov et al., 2015).

### Phylogenetic analysis

16S rRNA genes of strains were amplified using PCR and the universal bacterial primers 27F (5′-AGAGTTTGATCCTGGCTCAG-3′) and 1492R (5′-GGTTACCTTGTTACGACTT-3′). Purified PCR products were cloned into the vector pMD19-T (TaKaRa) and recombinant plasmids were transformed into *Escherichia coli* DH5α cells, followed by sequencing in Sangon Biotech (Shanghai, China). The 16S rRNA gene sequences of the isolates were compared with publicly available sequences in the EzBioCloud platform^2^ to determine the approximate taxonomic affiliations of the strains (Yoon et al., 2017a). Multiple sequence alignments and phylogenetic reconstructions of 16S rRNA genes were performed in MEGA (version 11) (Tamura et al., 2021). A phylogenetic tree was then inferred using neighbor-joining methods, and evolutionary distances were calculated using the Kimura 2-parameter substitution model (Shamsuzzaman et al., 2023). Maximum Parsimony and Maximum Likelihood phylogenetic methods were also used to evaluate the phylogenetic affiliations of the strains. The topologies of the resultant phylogenetic trees were evaluated using bootstrap analysis with 1,000 replicates (Deng et al., 2023).

### Genome sequencing and gene annotation

Genome sequencing was conducted on an Illumina HiSeq 4,000 system platform at the BGI sequencing company (Shenzhen, China). To prepare sequencing libraries, genomic DNA was randomly sheared to construct three read libraries with lengths of 300 bp using a Bioruptor ultrasonicator (Diagenode, Denville, NJ, United States) and physico-chemical methods. The paired-end fragment libraries were then sequenced on the Illumina platform. Low quality reads (those with consecutive bases covered by fewer than five reads) were discarded. The sequenced reads were then assembled using the SOAPdenovo (version 1.05) assembly software program (Li et al., 2010). Estimated completeness and contamination values for the genomes were estimated using the CheckM pipeline (Parks et al., 2015). Digital DNA–DNA hybridization (dDDH) and average nucleotide identity (ANI) values between the strains and related strains were calculated using the Genome-to-Genome Distance Calculator (GGDC, version 3.0)^3^ (Auch et al., 2010) and with the ezbiocloud platform (Yoon et al., 2017b), respectively. Genome-based phylogeny of supermatrix approach from protein sequences of the bac120 gene set (a collection of 120 single-copy protein sequences prevalent in bacteria) was constructed by using EasyCGTree version 3.0^4^ as described previously (Zhang et al., 2020). Evolutionary distances were calculated using the IQ-Tree software program (version 1.6.1) (Nguyen et al., 2015). The genome sequences of the strains of interest were downloaded from the NCBI genome database.^5^

Protein sequences encoded by the strains were predicted and annotated using the NCBI Prokaryotic Genome Annotation Pipeline (PGAP). Gene prediction was conducted for the genome assemblies using glimmer3^6^ with Hidden Markov models. tRNA, rRNA, and sRNA identification was conducted with tRNAscan-SE (Chan et al., 2021), RNAmmer (Lagesen et al., 2007), and the Rfam database (Gardner et al., 2009), respectively. Tandem repeat annotation was conducted using the Tandem Repeat Finder^7^, with minisatellite and microsatellite DNAs identified based on the numbers and lengths of repeat units. Functional annotation was also conducted based on the best hits within BLAST analyses. In addition, general functional annotations were identified by comparisons against several databases included the Kyoto Encyclopedia of Genes and Genomes (KEGG), Clusters of Orthologous Groups (COG), Non-Redundant Protein (NR), Swiss-Prot, Gene Ontology (GO), TrEMBL, and EggNOG databases. Predictions of gene clusters involved in natural product formation were conducted using antiSMASH (Blin et al., 2019).

### Pan-genome analysis

The bacterial pan-genome analysis (BPGA) pipeline (version 1.3) was used to assess the genomic diversity among *Mycobacterium* strains using default settings (Chaudhari et al., 2016). A total of 73 protein datasets were used for the pan-genome analysis, including for strains YC-RL4^T^, MB418^T^, HX176^T^, and 70 related mycobacterial strains that were previously classified as “*Mycolicibacterium*.” Orthologous gene/protein clusters (homologous families) were identified using the USEARCH clustering tool (Kim et al., 2014).

## Results and discussion

### Phenotypic properties of the novel strains

Cells of strains YC-RL4^T^, MB418^T^, and 205372^T^ were Gram-positive, non-motile and rod-shaped. The three isolates grew well on GYM and MB7H10 agar, with weak growth on NA and TSA agar. Colonies of strains YC-RL4^T^ and HX176^T^ grown on GYM medium and incubated for 48 h were 1.0–2.5 mm in diameter, opaque, ivory, and convex with a smooth surface. Colonies of strain HX176^T^ grown on GYM medium incubated for 48 h were 1.0–3.2 mm in diameter, dry, rough, and orange-colored with undulated/scalloped edges. All strains grew over the pH range of 6.0–7.0 (optimum: 7.0). Strain YC-RL4^T^ grew in the presence of 0–5.0% (w/v) NaCl (optimum: 0–3.0%, w/v), and 10–37°C (optimum: 28°C), while strain MB418^T^ grew at 0–5.0% (w/v) NaCl (optimum 0–3.0%, w/v), and 15–45°C (optimum: 28°C), and strain HX176^T^ grew in the presence of 0–4.0% NaCl (w/v) (optimum: 0–2.0%), and at 15–28°C (optimum: 28°C). All strains exhibited positive catalase reactions, but negative results for oxidase, starch hydrolysis, Voges–Proskauer, and H_2_S production tests. All strains exhibited positive enzymatic activities for alkaline phosphatase, esterase (C4), leucine arylamidase, lipase (C14), naphthol-AS-BI-phosphohydrolase, and valine arylamidase, based on API ZYM strip tests. None of the strains could produce acid from fermentation of amidon, amygdalin, arbutin, D-arabinose, D-ardonitol, D-cellobiose, D-fucose, D-galactose, D-lactose, D-maltose, D-mannitol, D-melezitose, D-melibiose, D-raffinose, D-sorbitol, D-trehalose, D-turanose, dulcitol, erythritol, gentiobiose, glycogen, inulin, L-arabitol, L-fucose, L-rhamnose, L-sorbose, L-xylose, mehyl-*α*-D-glucopyranoside, mehyl-*α*-D-mannopyranoside, mehyl-*β*-D-xylopyranoside, N-acetylglucosamine, potassium 5-ketogluconate, salicin, or xylitol. The major differentiating features among strains YC-RL4^T^, MB418^T^, HX176^T^, and other species are shown in Table 1.

**Table 1:** Physiological characteristics of strains YC-RL4^T^, MB418^T^, HX176^T^, and closely related *Mycobacterium* type strains.

Characteristic	1	2	3	4	5	6
NaCl tolerance (%, w/v)	0–5.0	0–5.0	0–5.0	0–5.0	0–4.0	0–3.0
Growth temperature range (°C)	10–37	4–37	15–37	15–45	15–28	10–45
Gelatin hydrolysis	−	−	−	−	+	−
Nitrate reduced to nitrite	+	+	+	−	−	+
Carbon sources used for growth						
D-trehalose	−	−	−	−	−	+
*α*-D-glucose	+	−	−	+	−	+
D-mannose	+	w	+	+	−	−
D-fructose	−	+	+	+	−	+
D-galactose	−	−	−	+	−	−
D-sorbitol	−	−	−	+	w	+
D-mannitol	−	+	+	+	w	+
D-arabitol	−	−	−	+	w	+
myo-inositol	−	+	+	+	w	−
Glycerol	+	+	+	w	−	w
D-fructose-6-PO_4_	w	+	w	−	w	w
Glycyl-L-proline	−	−	+	−	−	−
L-glutamic acid	+	+	+	−	−	−
D-gluconic acid	+	−	−	+	+	+
Quinic acid	−	+	−	+	w	−
D-saccharic acid	−	−	+	+	+	−
Methyl pyruvate	w	−	+	w	−	−
*α*-keto-glutaric acid	−	−	+	−	−	−
L-malic acid	+	+	+	−	−	+
Bromo-succinic acid	−	w	w	−	−	+
Tween 40	+	w	+	w	−	+
*α*-hydroxy-butyric acid	w	−	+	w	−	−
*β*-hydroxy-D,L-butyric Acid	+	+	+	−	+	+
*α*-keto-butyric acid	−	+	+	w	−	−
Formic acid	w	+	−	−	−	−
API ZYM results						
Cystine arylamidase	+	+	+	+	−	+
Trypsin	+	+	+	+	−	+
*α*-chymotrypsin	−	w	w	+	−	w
Acid phosphatase	+	+	+	+	−	+
*β*-galactosidase	−	+	−	−	−	−
*α*-glucosidase	+	+	+	+	−	+
*β*-glucosidase	−	+	+	+	−	+
Acid production from substrates (API 50CH)						
Glycerol	+	+	+	−	−	−
Erythritol	−	−	−	−	−	+
D-ribose	−	−	−	−	+	−
D-xylose	+	+	w	−	−	+
D-ardonitol	−	−	−	−	−	+
D-glucose	+	+	+	−	+	−
D-fructose	+	+	−	−	+	+
D-mannose	w	−	+	−	−	−
L-sorbose	−	−	−	−	−	+
L-rhamnose	−	−	−	−	−	+
Inositol	+	+	−	−	−	−
D-mannitol	−	+	−	−	−	+
D-sorbitol	−	−	−	−	−	+
D-cellobiose	−	−	−	−	−	+
D-trehalose	−	−	+	−	−	−
Gentiobiose	−	−	−	−	−	+
D-arabitol	−	−	−	+	−	+
L-arabitol	−	−	−	−	−	+

Strains: 1, YC-RL4^T^; 2, *M. fluoranthenivorans* JCM 14741^T^; 3. *M. frederiksbergense* DSM 44346^T^; 4, MB418^T^; 5, HX176^T^; 6, *M. litorale* DSM 45785^T^.

+, positive; w, weakly positive; −, negative activities/growth.

### Chemotaxonomic properties of strains

Strains YC-RL4^T^, MB418^T^, and HX176^T^ exhibited chemotaxonomic properties consistent with their classification as *Mycobacterium*. The strains produced whole organism hydrolysates rich in *meso*-diaminopimelic acid (*meso*-A_2_pm), arabinose, galactose, glucose, mannose, and ribose. MK-9 (H_2_) was the predominant menaquinone and the polar lipid profile contained diposphatidylglycerol (DPG), phosphatidylethanolamine (PE), and phosphatidylinositol (PI) (Supplementary Figure S1). The three strains nevertheless exhibited differences in several chemotaxonomic characteristics relative to other *Mycobacterium* strains. The fatty acid profile of strain YC-RL4^T^ comprised major levels of C_16:0_ (15.3%) and C_17:1_*ω*7*c* (15.3%), while the fatty acid profile of strain MB418^T^ exhibited major levels of C_17:1_*ω*7*c* (47.7%) and sum in feature 9 (iso- C_17:1_*ɷ9c*/C_16:0_ 10-methyl) (43.3%). Strain HX176^T^ exhibited higher levels of C_17:1_*ω*7*c* (43.2%) and C_19:1_ trans 7 (30.9%), but lower C_16:0_ levels (8.8%), while also lacking sum in feature 9 (Supplementary Table S1). In addition, only the polar lipid profiles of strains MB418^T^ and HX176^T^ included glycolipids (GLs), contrasting with that of strain YC-RL4^T^ that lacked GLs (Supplementary Figure S1). The HPLC profile of cell-wall mycolic acids from strain YC-RL4^T^, containing 60–70 carbon atoms of mycolic acids as the main peaks, was a little similar to that observed for *M. fluoranthenivorans* JCM 14741^T^ and *M. frederiksbergense* DSM 44346^T^ while quite different from that of strains MB418^T^, and HX176^T^. The HPLC pattern of cell-wall mycolic acids from strains MB418^T^ and HX176^T^ were more similar to that of *M. litorale* DSM 45785^T^ than others, and the chromatogram of strain MB418^T^ was distinct from that of strain HX176^T^ (Supplementary Figure S2).

### Phylogenetic analyses

**Figure 1:**
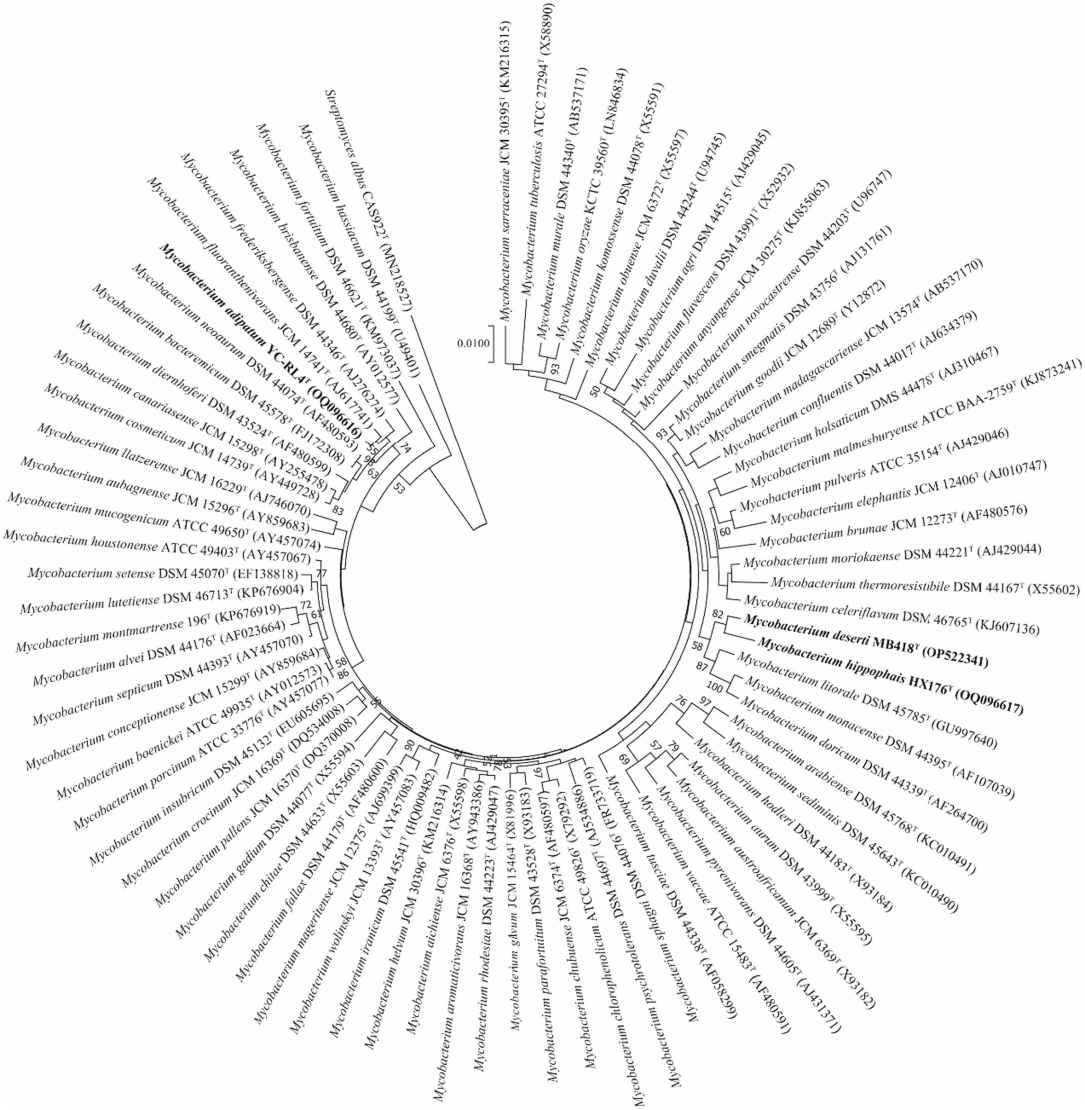
Neighbor-joining phylogenetic reconstruction of 16S rRNA gene sequences from strains YC-RL4^T^, MB418^T^, and HX176^T^ along with those from other *Mycobacterium*. Bootstrap values above 50% are shown as percentages of 1,000 replicates. The 16S rRNA gene sequence of *Streptomyces albus* CAS922^T^ (GenBank accession MN218527) was used as the outgroup. Scale bar indicates 0.01 nt substitutions per alignment site.

Nearly complete 16S rRNA gene sequences for strain YC-RL4^T^ (1,527 bp, accession OQ096616), MB418^T^ (1,509 bp, accession OP522341), and HX176^T^ (1,508 bp, accession OQ096617) were obtained. BLAST searches of the 16S rRNA gene sequences against the GenBank database indicated that strains YC-RL4^T^, MB418^T^, and HX176^T^ were closely related to *Mycobacterium* species of the family *Mycobacteriaceae* even though they were previously classified as the genus *Mycolicibacterium*. Strain YC-RL4^T^ was most closely related to *M. fluoranthenivorans* JCM 14741^T^ and *M. frederiksbergense* DSM 44346^T^, with 16S rRNA gene sequence similarities of 99.3 and 99.2%, respectively. The 16S rRNA gene of MB418^T^ exhibited 98.5% nucleotide similarity to 16S rRNA genes of *M. celeriflavum* DSM 46765^T^ and *M. moriokaense* DSM 44221^T^, but with 97.2% similarity to the 16S rRNA genes of strain YC-RL4^T^, and 96.2–98.3% similarity to 16S rRNA genes of other various *Mycobacterium* type strains. The 16S rRNA gene sequence of strain HX176^T^ exhibited a nucleotide similarity of 98.3% to that of *M. litorale* DSM 45785^T^, 98.0% to that of strain YC-RL4^T^, and similarities of 95.8–97.9% to those from other *Mycobacterium* type strains (Supplementary Table S2). Phylogenetic analysis of the 16S rRNA gene sequences of the three isolates with other mycobacterial strains that were previously classified as “*Mycolicibacterium*” revealed that the strains belonged to the genus *Mycobacterium*. The 16S rRNA gene sequence of strain YC-RL4^T^ formed a sub-clade with that of the type strain *M. fluoranthenivorans* JCM 14741^T^, which were together related to the 16S rRNA gene of *M. frederiksbergense* DSM 44346^T^, consistent with BLAST comparisons. The 16S rRNA gene of strain MB418^T^ formed a well-supported sub-clade with the 16S rRNA gene of strain HX176^T^, which were together associated with that of *M. litorale* DSM 45785^T^ (Figure 1). The whole-genome phylogenomic analysis recapitulated the 16S rRNA gene phylogenetic analysis, with the exception of the strain YC-RL4^T^ genome being most closely related to that of *M. frederiksbergense* DSM 44346^T^ (Supplementary Figure S3), contrasting with the 16S rRNA gene phylogenetic analysis.

### Genomic characteristics

The genomes of strains YC-RL4^T^, MB418^T^, and HX176^T^ were estimated to be 99.95, 100.00, and 100.00% complete, with estimated contamination of 0.62, 0.30, and 0.45%, respectively. The draft genome sizes of strains YC-RL4^T^, MB418^T^, and HX176^T^ were 6.1, 5.6, and 5.9 Mbp, respectively, and were assembled from two contigs with an N50 length of 5,801,417 bp; 15 contigs with an N50 length of 710,882 bp; and 41 contigs with an N50 length of 302,245 bp, respectively (Supplementary Table S3). The genomic sequence for strain YC-RL4^T^ encoded 5,881 total genes including 47 tRNA, 6 rRNA, 4 other ncRNA, and 83 pseudo genes. The genome of strain MB418^T^ encoded 5,444 total genes including 47 tRNA, 3 rRNA, 3 other ncRNA, and 98 pseudo genes. The genome of strain HX176^T^ encoded 5,692 genes including 46 tRNA, 4 rRNA, 3 other ncRNA, and 64 pseudo genes. The genomic G + C content of all three strains ranged between 66.5 and 69.3%. ANI values calculated between strains YC-RL4^T^, MB418^T^, HX176^T^, and other *Mycobacterium* species were all <86.9%, with corresponding dDDH values all <31.8% (Supplementary Table S4). The values were lower than the thresholds used to delineate bacterial species (i.e., ANI < 95–96% and dDDH <70%) (Kim et al., 2014), thereby indicating that the strains represented novel, uncharacterized species.

### Functional gene complements

Polycyclic aromatic hydrocarbons ring-hydroxylating dioxygenases (PAH-RHDs) are critical for PAH degradation by aerobic bacteria, because they catalyze the initial oxidation of O_2_ atoms to form cisdihydrodiol and this step controls the PAH degradation rate (Kim et al., 2007; Chemerys et al., 2014). Among PAH-RHDs, aromatic ring-hydroxylating dioxygenases are particularly important in the aerobic bacterial degradation of aromatic compounds, because they catalyze the oxidation of the compounds during degradation. They typically catalyze the addition of two hydroxy groups to vicinal carbons, thereby disrupting aromaticity and yielding dihydrodiol compounds with *cis*, *cis* stereochemistry. The enzymes are biotechnologically important because they act as biocatalysts in the stereospecific synthesis of chiral synthons and the degradation of aromatic pollutants (Kahl and Hofer, 2003). Multiple genes encoding dioxygenases were identified in the genomes of strains YC-RL4^T^, MB418^T^, and HX176^T^. Specifically, four genes putatively encoding alpha subunits with homology to aromatic ring-hydroxylating dioxygenases were encoded by the genome of strain YC-RL4^T^, while genes putatively encoding alpha subunits of aromatic ring-hydroxylating dioxygenases were identified in the genomes of strains MB418^T^ and HX176^T^ (Supplementary Table S5).

Strains of the genus *Mycobacterium* could degrade PAHs to central intermediates via the *o*-phthalate and the *β*-ketoadipate pathway (Kim et al., 2007; Augelletti et al., 2020). The *o*-phthalate degradation pathway of Gram-positive bacteria involves oxygenation to form 3,4-dihydro-3,4-dihydroxyphthalate, dehydrogenation to 3,4-dihydroxyphthalate, and finally decarboxylation to generate protocatechuate (Bhattacharyya et al., 2023). Genes (*phtAa*) were identified that encoded homologous proteins involved in the degradation of phthalate in the genomes of strains YC-RL4^T^, MB418^T^, and HX176^T^ (Supplementary Table S5). Thus, these three strains could likely degrade various types of phthalates, as previously suggested. In addition, genes of the *β*-ketoadipate pathway cluster (*pcaC*, *pcaD*, *pcaG*, and *pcaH*) were also identified in the genomes of strains YC-RL4^T^, MB418^T^, and HX176^T^ (Supplementary Table S5), suggesting that anthracene degradation by these strains could proceed through the ortho-cleavage of protocatechuate (protocatechuic acid [PCA] 3,4-dioxygenase) (Habe et al., 2005). These protocatechuate catabolic genes were required for the complete degradation of PAHs to TCA cycle intermediates. Consequently, strains YC-RL4^T^, MB418^T^, and HX176^T^ exhibited the potential for the bioremediation of PAHs and smaller ring aromatics.

Although heavy metals naturally exist in many environments (Masindi and Muedi, 2018), the ubiquitous distribution of heavy metals in natural environments is a consequence of global industrialization and urbanization that has led to negative impacts on human and environmental health. Microorganisms have adapted multiple resistance mechanisms to overcome the physiological stress from heavy metal exposure (Sherpa et al., 2020). Several genes were identified in the genomes of strains YC-RL4^T^, MB418^T^, and HX176^T^ that encoded heavy metal resistance proteins. For example, the three strains exhibited the potential to mediate copper toxicity via copper-resistant genes (*copC*) identified in all of their genomes. Further, the copper-resistance gene *copD* was also identified in the genomes of strain MB418^T^ and HX176^T^. Free form copper is highly toxic due to its ability to produce radicals during cycling between oxidized Cu(II) and reduced Cu(I) forms. Consequently, intracellular copper must remain complexed within a tightly controlled copper homeostatic system. Importantly, Cop proteins sequester excess copper in periplasms and outer membranes (Arnesano et al., 2003). Consequently, these proteins might confer copper resistance to strains YC-RL4^T^, MB418^T^, and HX176^T^ by helping to sequester and accumulate copper in periplasms with copper binding proteins, thereby preventing toxic levels of copper from entering the cytoplasm.

In addition to the above, genes (*merA* and *merB*) that encoded mercury reductase enzymes were identified in the strain YC-RL4^T^ genome that enable resistance to mercury stress. Mercury toxicity arises from the strong affinity of monomethyl-Hg and Hg^2+^ to sulfur atoms in cysteine residues and, hence, interference with protein structure and function. The mercury reductase encoded by *merA* and *merB* reduced the toxicity of Hg2+ to Hg^0^ (Møller et al., 2014). Specifically, organomercurial lyase (MerB) catalyzed the protonolysis of the carbon-mercury bond, resulting in the formation of ionic mercury and reduced hydrocarbon. The ionic mercury (Hg^2+^) was subsequently reduced to less reactive elemental mercury (Hg^0^) by mercuric reductase (MerA) (Di Lello et al., 2004). The presence of these genes involved in PAH-degradation and metal resistance genes in the genomes of these strains suggested that strains YC-RL4^T^, MB418^T^, and HX176^T^ could be applied in bioremediation of soils contaminated with both PAHs and heavy metals.

### Pangenomic analyses

**Figure 2:**
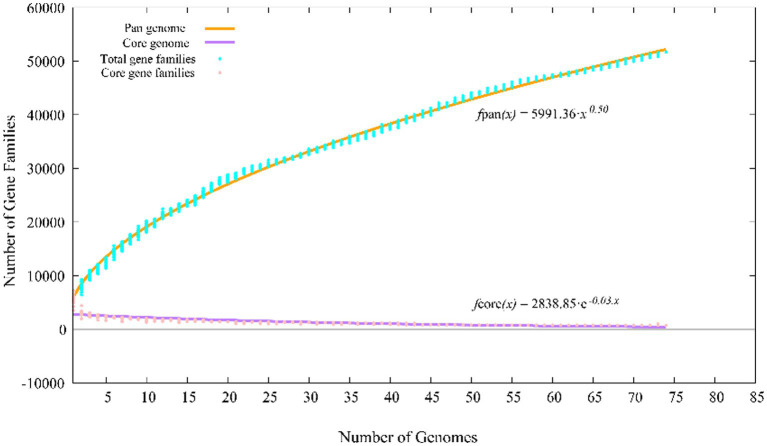
Pan-genome profiles of strains YC-RL4^T^, MB418^T^, HX176^T^ and 70 related *Mycobacterium* strains based on protein clustering.

The pan-genomes of strains comprise the entire gene repertoire for a given species across several populations, and their analysis can be informative for understanding the distribution of core, important genes shared by all strains of a species, in addition to strain-specific accessory genes that may be more dispensable (Reis and Cunha, 2021). A total of 401,362 protein-coding genes (Table 2) were encoded by the genomes of strains YC-RL4^T^, MB418^T^, HX176^T^, and 70 other related strains of the genus *Mycobacterium*, comprising 51,712 homologous families based on cluster analysis. A total of 734 core genes were shared by the 73 strains and comprise the core genome identified in this study. The core genes accounted for approximately 13.5% of the pan-genome for the 73 strains of the genus *Mycobacterium*. The identification of the “open” pangenome based on consideration of increasing numbers of strains was fit with a powerlaw regression function [f(X) = 5991.36×^0.50^], while the core genome identification fit an exponential regression [f(X) = 2838.85e^−0.03X^; Figure 2]. Thus, the relatively extensive open pangenome of the strains indicates that they have considerable repertoires of accessory genes, possibly due to extensive horizontal gene exchanges with various microbial species (Coyte et al., 2022). Aromatic ring-hydroxylating dioxygenase alpha subunit-encoding genes were identified in all of the 73 genomes, suggesting it was an important core gene shared by all strains and plays a critical role in PAH degradation by the strains. In addition, the copy numbers of these genes varied among different strains. The lowest number of copies was observed in the genome of *M. brumae* DSM 44177^T^, while the largest number of copies was observed in the genome of strain *M. gadium* DSM 44077^T^.

**Table 2:** Pan-genome information for *Mycobacterium* strains evaluated in this study, including strains YC-RL4^T^, MB418^T^, HX176^T^, and 70 others.

GenomeID	Organism name	No. of core genes	No. of accessory genes	No. of unique genes	No. of exclusively absent genes
1	HX176^T^	734	4,190	505	1
2	YC-RL4^T^	734	4,432	266	0
3	MB418^T^	734	4,101	494	4
4	*Mycobacterium agri* JCM 6377^T^	734	4,399	935	5
5	*Mycobacterium aichiense* DSM 44147^T^	734	4,496	176	1
6	*Mycobacterium alvei* JCM 12272^T^	734	4,245	365	5
7	*Mycobacterium anyangense* JCM 30275^T^	734	3,827	435	0
8	*Mycobacterium arabiense* DSM 45768^T^	734	4,536	445	1
9	*Mycobacterium aromaticivorans* JCM 16368^T^	734	4,511	265	1
10	*Mycobacterium aubagnense* JCM 15296^T^	734	4,297	497	0
11	*Mycobacterium aurum* NCTC 10437^T^	734	4,495	164	2
12	*Mycobacterium austroafricanum* DSM 44191^T^	734	4,836	333	0
13	*Mycobacterium bacteremicum* DSM 45578^T^	734	4,441	225	0
14	*Mycobacterium boenickei* JCM 15653^T^	734	5,063	129	1
15	*Mycobacterium brisbanense* DSM 44680^T^	734	5,304	617	2
16	*Mycobacterium brumae* DSM 44177^T^	734	2,383	476	27
17	*Mycobacterium canariasense* DSM 44828^T^	734	5,050	567	1
18	*Mycobacterium celeriflavum* DSM 46765^T^	734	3,610	144	1
19	*Mycobacterium chitae* JCM 12403^T^	734	3,700	366	8
20	*Mycobacterium chlorophenolicum* DSM 43826^T^	734	5,165	495	2
21	*Mycobacterium chubuense* DSM 44219^T^	734	4,542	103	3
22	*Mycobacterium conceptionense* CCUG 50187^T^	734	4,820	264	0
23	*Mycobacterium confluentis* DSM 44017^T^	734	4,021	394	1
24	*Mycobacterium cosmeticum* DSM 44829^T^	734	5,020	174	0
25	*Mycobacterium crocinum* DSM 45433^T^	734	4,523	129	1
26	*Mycobacterium diernhoferi* DSM 43524^T^	734	4,275	297	1
27	*Mycobacterium duvalii* DSM 44244^T^	734	3,967	238	0
28	*Mycobacterium elephantis* DSM 44368^T^	734	4,002	148	2
29	*Mycobacterium fallax* DSM 44179^T^	734	2,628	460	8
30	*Mycobacterium flavescens* DSM 43991^T^	734	4,370	261	0
31	*Mycobacterium fluoranthenivorans* JCM 14741^T^	734	4,452	422	25
32	*Mycobacterium fortuitum* JCM 6387^T^	734	4,686	245	1
33	*Mycobacterium frederiksbergense* DSM 44346^T^	734	4,688	213	3
34	*Mycobacterium gadium* DSM 44077^T^	734	4,820	220	2
35	*Mycobacterium gilvum* DSM 45363^T^	734	4,478	175	1
36	*Mycobacterium goodii* ATCC 700504^T^	734	5,039	250	0
37	*Mycobacterium hassiacum* DSM 44199^T^	734	3,307	392	15
38	*Mycobacterium helvum* JCM 30396^T^	734	4,486	353	1
39	*Mycobacterium hodleri* DSM 44183^T^	734	4,628	543	1
40	*Mycobacterium holsaticum* DSM 44478^T^	734	3,917	278	0
41	*Mycobacterium insubricum* DSM 45132^T^	734	2,195	703	250
42	*Mycobacterium iranicum* DSM 45541^T^	734	4,706	229	0
43	*Mycobacterium komossense* DSM 44078^T^	734	4,482	887	2
44	*Mycobacterium litorale* DSM 45785^T^	734	4,159	176	0
45	*Mycobacterium llatzerense* DSM 45343^T^	734	4,445	626	1
46	*Mycobacterium lutetiense* DSM 46713^T^	734	4,280	379	14
47	*Mycobacterium madagascariense* DSM 45167^T^	734	3,818	506	0
48	*Mycobacterium mageritense* JCM 12375^T^	734	5,798	640	1
49	*Mycobacterium malmesburyense* CIP 110822^T^	734	4,038	145	3
50	*Mycobacterium monacense* JCM 15658^T^	734	4,462	186	1
51	*Mycobacterium moriokaense* JCM 6375^T^	734	4,520	352	2
52	*Mycobacterium mucogenicum* DSM 44124^T^	734	4,528	371	0
53	*Mycobacterium murale* DSM 44340^T^	734	4,523	810	1
54	*Mycobacterium neoaurum* DSM 44074^T^	734	4,022	197	1
55	*Mycobacterium novocastrense* DSM 44203^T^	734	4,560	330	0
56	*Mycobacterium obuense* DSM 44075^T^	734	4,213	215	4
57	*Mycobacterium pallens* JCM 16370^T^	734	4,558	157	1
58	*Mycobacterium parafortuitum* ATCC 19686^T^	734	4,539	165	0
59	*Mycobacterium porcinum* ATCC 33776^T^	734	5,320	165	3
60	*Mycobacterium psychrotolerans* DSM 44697^T^	734	4,170	226	3
61	*Mycobacterium pulveris* DSM 44222^T^	734	3,992	281	0
62	*Mycobacterium pyrenivorans* DSM 44605^T^	734	4,520	364	2
63	*Mycobacterium rhodesiae* DSM 44223^T^	734	4,566	138	0
64	*Mycobacterium sarraceniae* JCM 30395^T^	734	3,054	359	3
65	*Mycobacterium sediminis* DSM 45643^T^	734	4,418	448	0
66	*Mycobacterium septicum* DSM 44393^T^	734	4,930	235	11
67	*Mycobacterium setense* DSM 45070^T^	734	4,702	166	0
68	*Mycobacterium smegmatis* NCTC 8159^T^	734	5,069	417	0
69	*Mycobacterium sphagni* DSM 44076^T^	734	4,269	526	0
70	*Mycobacterium thermoresistibile* DSM 44167^T^	734	3,092	467	12
71	*Mycobacterium tusciae* DSM 44338^T^	734	4,639	182	0
72	*Mycobacterium vaccae* NBRC 14118^T^	734	4,564	233	3
73	*Mycobacterium wolinskyi* JCM 13393^T^	734	5,598	562	0

**Figure 3:**
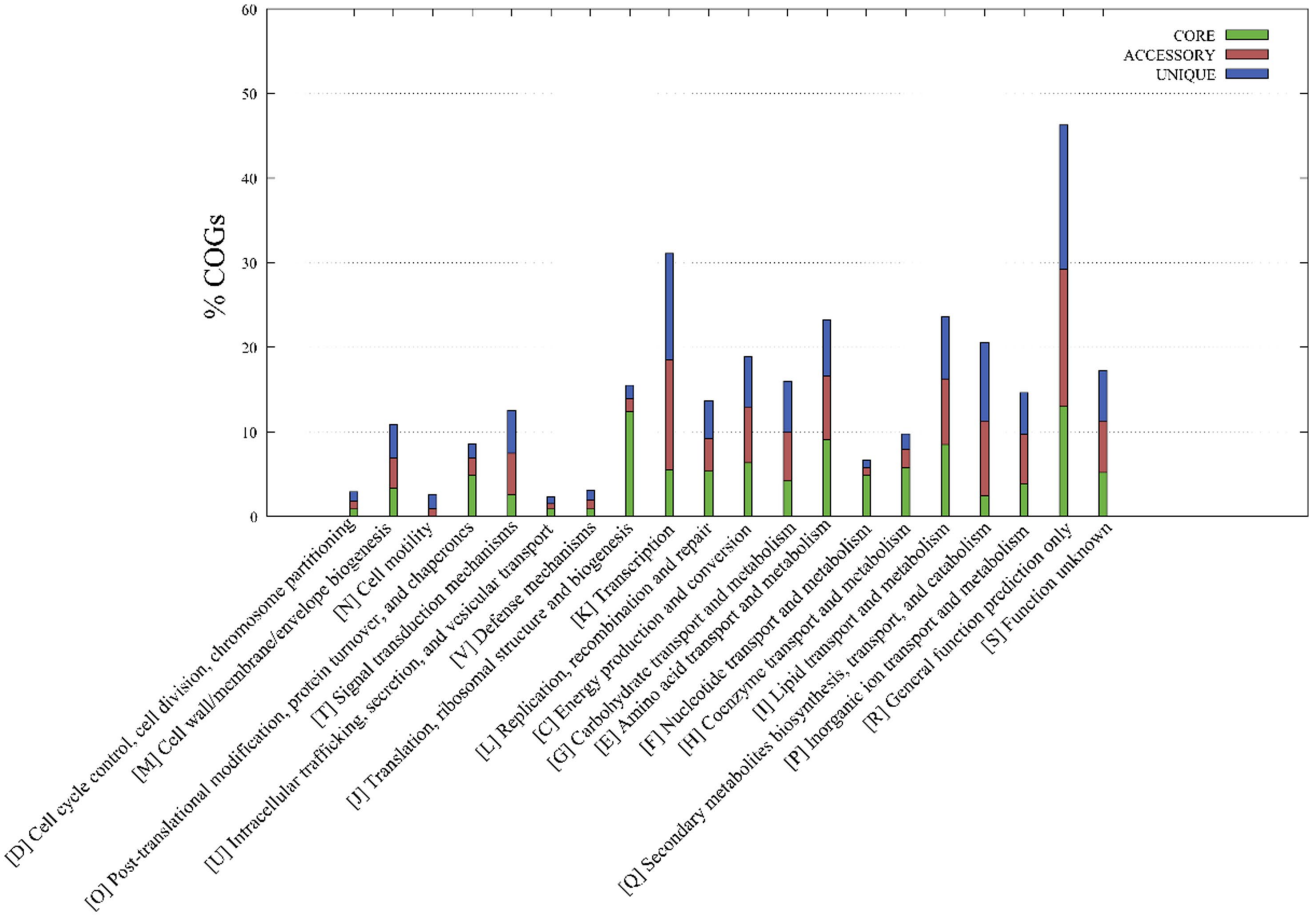
Metabolic pathways associated with the core, accessory, and unique genes of strains YC-RL4^T^, MB418^T^, HX176^T^, and 70 other related *Mycobacterium* strains. The metabolic annotations are based on clusters of orthologous genes (COG) database annotations.

A functional classification of core, accessory, and unique genes was performed based on comparison to the COG database, revealing clear differences between core and accessory genes. Core genes were most associated with the COG categories of R (general function prediction only; 13.0% of genes), J (translation, ribosomal structure, and biogenesis; 12.4%), E (amino acid transport and metabolism; 9.1%) and I (lipid transport and metabolism; 8.2%) (Figure 3), while accessory and unique genes were most associated with the COG categories R (general function prediction only; 16.3 and 17.0%, respectively), K (transcription; 13.0 and 12.6%), and Q (secondary metabolite biosynthesis, transport, and catabolism; 8.8 and 9.3%). Thus, core genes were mostly involved in basic physiological functions, resulting in the overall prevailing phenotypes of the strains. In contrast, accessory genes related to tachytely evolution could increase the gene and functional diversity of their respective genomes. These accessory genes may be involved in metabolic pathways that critically ensure adaptations or functioning in variable ecological niches that differ among species (Vernikos et al., 2015).

### Secondary metabolite biosynthesis gene cluster analysis

Polyketide synthesis operons and others involved in non-ribosomal peptide synthesis (NRPS) are common in the genomes of *Mycobacterium* and are responsible producing cell wall-associated lipids, siderophores, and other biologically active molecules (Stinear et al., 2008). AntiSMASH analysis revealed the presence of 14–21 biosynthetic gene clusters (BGCs) in the genomes of strain YC-RL4^T^, MB418^T^, and HX176^T^ that were especially enriched in genes encoding Type I polyketide synthases (PKSs) and non-ribosomal peptide synthases (NRPSs) (Supplementary Table S6). A BGC that was most similar to that encoding mycobactin was identified in all 73 genomes, consistent with the production of mycobactin being essential for *Mycobacterium* strains to access iron (McMahon et al., 2012). In addition, several putative BGCs exhibited low similarities to known clusters, indicating their potential for synthesizing putatively novel secondary metabolites.

## Conclusion

In this study, three strains of the genus *Mycobacterium* (YC-RL4^T^, MB418^T^, and HX176^T^) were isolated from several petroleum-contaminated soil samples of China. The three strains exhibited highest similarity to *Mycobacterium* species of the family *Mycobacteriaceae*. The 16S rRNA gene sequence of strain YC-RL4^T^ in comparison to other characterized *Mycobacterium* strains exhibited levels below the threshold for differentiating species (98.65%), while the 16S rRNA gene sequences of strains MB418^T^and HX176^T^ compared to other characterized *Mycobacterium* strains were above 98.65%. However, the dDDH values among the three strains and the other characterized *Mycobacterium* species were very below the threshold value (70%) used to delineate bacterial strains of the same species (46). Moreover, the ANI values among all three strains and other type species of *Mycobacterium* were much lower than the threshold for bacterial species delineation (95–96%) (Kim et al., 2014). Thus, strains YC-RL4^T^, MB418^T^, and HX176^T^ likely represent three novel species of *Mycobacterium*. Chemotaxonomic, genomic, and phenotypic analyses of strains YC-RL4^T^, MB418^T^, and HX176^T^ confirmed their identification as novel species. Lastly, the genomes of strains YC-RL4^T^, MB418^T^, and HX176^T^ encoded proteins associated with PAH degradation and metal resistance, consistent with strain YC-RL4^T^ previously being shown to degrade phthalates (Ren et al., 2016). Consequently, these three species may hold considerable application potential in the bioremediation of PAH and metal-contaminated soils.

### Description of *Mycobacterium adipatum* sp. nov.

(a.di.pa’tum. L. neut. adj. *adipatum*, fat, greasy)

Cells are Gram-positive, non-motile, and rod-shaped, producing opaque, ivory, and convex colonies on GYM and MB7H10 agar after cultivation at 48 h and 28°C. Cells are catalase-negative and oxidase-positive. Optimum growth was observed at 10–37°C, pH 6–7, and with NaCl concentrations ranging between 0 and 5%. Cells were negative for Voges–Proskauer, H_2_S production, and starch hydrolysis tests. Acetic acid, D-gluconic acid, D-mannose, glucuronamide, glycerol, L-glutamic acid, L-malic acid, propionic acid, tween 40, *α*-D-glucose, *β*-hydroxy-D, and L-butyric acid can be utilized as carbon sources. 3-methyl glucose, acetoacetic acid, bromo-succinic acid, citric acid, D-arabitol, D-aspartic acid, D-cellobiose, dextrin, D-fructose, D-fucose, D-galactose, D-galacturonic acid, D-glucose-6-PO_4_, D-glucuronic acid, D-lactic acid methyl ester, D-maltose, D-mannitol, D-melibiose, D-raffinose, D-saccharic acid, D-salicin, D-serine, D-sorbitol, D-trehalose, D-turanose, gelatin, gentiobiose, glycyl-L-proline, inosine, L-aspartic acid, L-fucose, L-galactonic acid lactone, L-histidine, L-pyroglutamic acid, L-rhamnose, L-serine, mucic acid, myo-inositol, N-acetyl neuraminic acid, N-acetyl-D-galactosamine, N-acetyl-D-glucosamine, N-acetyl-*β*-D-mannosamine, pectin, p-hydroxy-phenylacetic acid, quinic acid, stachyose, sucrose, *α*-D-lactose, *α*-keto-butyric acid, *α*-keto-glutaric acid, *β*-methyl-D-glucoside, and *γ*-amino-butryric acid are not used as carbon sources. D-fructose, D-glucose, D-xylose, esculin ferric citrate, glycerol, inositol, potassium 2-ketogluconate, and potassium gluconate can be assimilated and fermented to produce acid. Cells exhibit positive acid phosphatase, alkaline phosphatase, cystine arylamidase, esterase (C4), esterase lipase (C8), leucine arylamidase, lipase (C14), naphthol-AS-BI-phosphohydrolase, trypsin, valine arylamidase, and *α*-glucosidase activities *via* API ZYM strip assays. Whole-cell hydrolysates contain *meso*-diaminopimelic acid (*meso*-A_2_pm), arabinose, galactose, glucose, mannose, and ribose. Cellular polar lipid profiles comprise diposphatidylglycerol (DPG), phosphatidylethanolamine (PE) and phosphatidylinositol (PI). The primary menaquinone is MK-9 (H_2_). The major fatty acids were C_16:0_ (15.3%) and C_17:1_*ω*7*c* (15.3%). The mycolic acids primarily contain 60–70 carbon atoms. The type strain YC-RL4^T^ (= CPCC 205684^T^ = CGMCC 1.62027^T^) was isolated from a petroleum-contaminated soil sample from Shandong, China. The draft genome of the type strain is 6.1 Mbp in size and exhibits a genomic G + C content of 67.4%.

### Description of *Mycobacterium deserti* sp. nov.

(de.ser’ti. L. gen. n. *deserti*, from a desert)

Cells are Gram-positive, non-motile, and non-spore forming. Ivory colonies develop on GYM and MB7H10 agar after incubation for 48 h at 28°C. Optimal growth was observed at 15–45°C, pH 6–7, and NaCl concentrations ranging from 0 to 5%. Cells were oxidase-positive and catalase-negative, but exhibited negative results for Voges–Proskauer, H_2_S production, and starch hydrolysis tests. Cells can utilize acetic acid, D-arabitol, D-fructose, D-galactose, D-gluconic Acid, D-mannitol, D-mannose, D-saccharic acid, D-sorbitol, myo-inositol, propionic acid, quinic acid, and α-D-glucose as sole carbon sources. API 50CH tests indicated that arbutin, D-lyxose, D-saccharose, D-tagatose, esculin ferric citrate, potassium 2-ketogluconate, and potassium gluconate are used as carbon sources. In contrast, amidon, amygdalin, D-arabinose, D-arabitol, D-ardonitol, D-cellobiose, D-fructose, D-fucose, D-galactose, D-glucose, D-lactose, D-maltose, D-mannitol, D-mannose, D-melezitose, D-melibiose, D-raffinose, D-ribose, D-sorbitol, D-trehalose, D-turanose, dulcitol, D-xylose, erythritol, gentiobiose, glycerol, glycogen, inositol, inulin, L-arabinose, L-arabitol, L-fucose, L-rhamnose, L-sorbose, L-xylose, mehyl-*α*-D-glucopyranoside, mehyl-*α*-D-mannopyranoside, Mehyl-*β*-D-xylopyranoside, N-acetylglucosamine, potassium 5-ketogluconate, salicin, and xylitol are not used as carbon sources. API ZYM analysis revealed the presence of positive acid phosphatase, alkaline phosphatase, cystine arylamidase, esterase (C4), leucine arylamidase, lipase (C14), naphthol-AS-BI-phosphohydrolase, trypsin, valine arylamidase, *α*-chymotrypsin, *α*-glucosidase, and *β*-glucosidase activities. Whole-cell hydrolysates contained *meso*-diaminopimelic acid (*meso*-A_2_pm), arabinose, galactose, glucose, mannose, and ribose. Cellular polar lipid profiles comprised diposphatidylglycerol (DPG), phosphatidylethanolamine (PE), phosphatidylinositol (PI), and glycolipids (GL). The primary menaquinone was MK-9 (H_2_). The major fatty acids were C_17:1_*ω*7*c* (47.7%) and summed feature 9 (iso- C_17:1_*ɷ9c*/C_16:0_ 10-methyl) (43.3%). The mycolic acids mainly contain 78–86 carbon atoms, with moderate amount of 76–84 carbon atoms, and minor of 62–70 carbon atoms. The type strain MB418^T^ (= CPCC 205710^T^ = KCTC 49782^T^) was isolated from a gravel soil sample from the Gurbantunggut desert, China. The draft genome of the type strain is 5.6 Mbp in length and exhibits a genomic G + C content of 66.5%.

### Description of *Mycobacterium hippophais* sp. nov.

(hip.po.pha’is. L. gen. n. *hippophais*, from *Hippophae*, a plant genus whose rhizosphere the type strain was isolated from)

Cells are Gram-positive, non-motile, and rod-shaped, producing yellow colonies. Cells were positive for oxidase activity, but negative for catalase, Voges–Proskauer, H_2_S production, and starch hydrolysis tests. Optimum growth was observed at 15–28°C, pH 6–7, and with NaCl concentrations ranging between 0 and 4%. Acetic acid, D-gluconic acid, D-saccharic Acid, propionic acid, and β-hydroxy-D,L-butyric acid can be used as substrates. API 50CH analysis revealed that D-fructose, D-glucose, D-ribose, esculin ferric citrate, potassium 2-ketogluconate, and potassium gluconate can be used as carbon substrates. Enzymatic activity analysis revealed the presence of alkaline phosphatase, esterase (C4), esterase lipase (C8), leucine arylamidase, lipase (C14), naphthol-AS-BI-phosphohydrolase, and valine arylamidase activities, but the lack of activity for acid phosphatase, cystine arylamidase, N-acetyl-β-glucosamimidase, trypsin, *α*-chymotrypsin, *α*-fucosidase, *α*-galactosidase, *α*-glucosidase, *α*-mannosidase, *β*-galactosidase, *β*-glucosidase, and *β*-glucuronidase. Whole-cell hydrolysates contained *meso*-diaminopimelic acid (*meso*-A_2_pm), arabinose, galactose, glucose, mannose, and ribose. Cellular polar lipid profiles comprised diposphatidylglycerol (DPG), phosphatidylethanolamine (PE), phosphatidylinositol (PI), and glycolipids (GL). The primary menaquinone was MK-9 (H_2_). The major fatty acids were C_17:1_*ω*7*c* (43.2%), C_19:1_ trans 7 (30.9%), and C_16:0_ (8.8%). The mycolic acids primarily contain 68–78 carbon atoms. The type strain HX176^T^ (= CPCC 205372^T^ = KCTC 49413^T^) was isolated from a rhizosphere soil sample of a medicinal plant in Xinjiang, China. The draft genome of the type strain is 5.9 Mbp in length and exhibits a genomic G + C content of 69.3%.

The 16S rRNA gene sequences of strains YC-RL4^T^, MB418^T^, and HX176^T^ were deposited in GenBank under the accession numbers OQ096616, OP522341 and OQ096617, respectively. The whole genome shotgun data for the genomes of strains YC-RL4^T^, MB418^T^, and HX176^T^ were deposited in the DDBJ, ENA, and GenBank databases under the accession numbers CP015596, JAODWD000000000, and JAPZPY000000000, respectively.

## Data availability statement

The datasets presented in this study can be found in online repositories. The names of the repository/repositories and accession number(s) can be found in the article/Supplementary material.

## Author contributions

YD, TM, JW, and JS carried out the experiments. YD, YY, and Y-QZ conceived the research, analyzed the data, and prepared the manuscript. All authors contributed to the article and approved the submitted version.

## Funding

This research was supported by CAMS Innovation Fund for Medical Sciences (CIFMS, 2021-I2M-1-055), National Natural Science Foundation of China (32170021 and 81960712), Beijing Natural Science Foundation (5212018), Key project at central government level-the ability establishment of sustainable use for valuable Chinese medicine resources (2060302), and the National Infrastructure of Microbial Resources (NIMR-2021-3).

## Conflict of interest

The authors declare that the research was conducted in the absence of any commercial or financial relationships that could be construed as a potential conflict of interest.

## Publisher’s note

All claims expressed in this article are solely those of the authors and do not necessarily represent those of their affiliated organizations, or those of the publisher, the editors and the reviewers. Any product that may be evaluated in this article, or claim that may be made by its manufacturer, is not guaranteed or endorsed by the publisher.

## Footnotes

^1^ https://lpsn.dsmz.de/genus/mycobacterium

^2^ http://www.ezbiocloud.net/identify

^3^ http://ggdc.dsmz.de/ggdc.php

^4^ https://github.com/zdf1987/EasyCGTree

^5^ https://www.ncbi.nlm.nih.gov/genome/

^6^ http://www.cbcb.umd.edu/software/glimmer/

^7^ http://tandem.bu.edu/trf/trf.html

## Abbreviations

DPG: diphosphatidylglycerol
PE: phosphatidylethanolamine
PI: phosphatidylinositol
ANI: average nucleotide identity
dDDH: digital DNA–DNA hybridization
PAHs: polycyclic aromatic hydrocarbons
PAH-RHDs: polycyclic aromatic hydrocarbons ring-hydroxylating dioxygenases
