# Supplementary material for: Characterization of three rapidly growing novel Mycobacterium species with significant polycyclic aromatic hydrocarbon bioremediation potential
Source: Front Microbiol. 2023 Sep 8;14:1225746. doi: 10.3389/fmicb.2023.1225746 (PMC10517868; doi:10.3389/fmicb.2023.1225746)
Supplement: Supplementary file 1 [file Data_Sheet_1.PDF]

Characterization of three rapidly growing novel *Mycobacterium* species with significant polycyclic aromatic hydrocarbon bioremediation potential

Yang Deng[1,2†], Tong Mu[1,2†], Junhuan Wang[3†], Jing Su[1], Yanchun Yan[3*], Yu-Qin Zhang[1,2*]

[1]Institute of Medicinal Biotechnology, Chinese Academy of Medical Sciences & Peking Union Medical College, Beijing 100050, China

[2]State Key Laboratory of Dao-di Herb, Beijing, 100700, China

[3]Graduate School of Chinese Academy of Agricultural Sciences, Beijing 100081, China

[*]Author for correspondence:

Yanchun Yan

Email: yanyanchun@caas.cn

Yu-Qin Zhang

Email: yzhang@imb.pumc.edu.cn

Tel: +86-10-83167110

Fax: +86-10-83167110

Keywords: ***Mycobacterium adipatum*, *Mycobacterium deserti*, *Mycobacterium hippophais*, genome, bioremediation potential**

Abbreviations: DPG, diphosphatidylglycerol; PE, phosphatidylethanolamine; PI, phosphatidylinositol; ANI, average nucleotide identity; dDDH, digital DNA-DNA hybridization; PAHs, polycyclic aromatic hydrocarbons; PAH-RHDs, polycyclic aromatic hydrocarbons ring-hydroxylating dioxygenases.

Supplementary Figure S1. Polar lipid profiles of strains YC-RL4^T, MB418^T, and HX176^T after separation by two-dimensional thin layer chromatography.

Detection is shown after spraying with (a) molybdatophosphoric acid reagent; (b) ninhydrin stain reagent (c); molybdenum blue stain reagent; and (d) p-anisaldehyde stain reagent.

DPG, diphosphatidylglycerol; PE, phosphatidylethanolamine; PI, phosphatidylinositol; GL, glycolipid

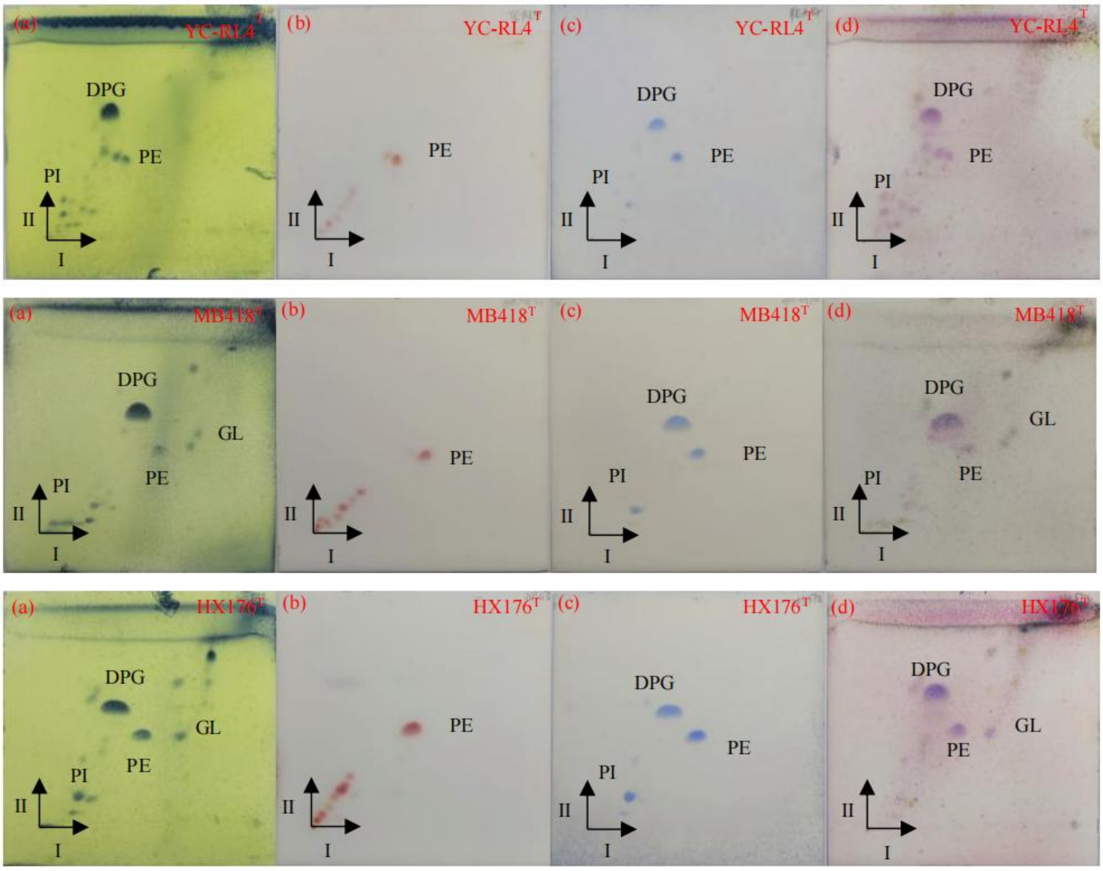

Supplementary Figure S2. HPLC profiles of mycolic acids of strains YC-RL4^T, MB418^T, and HX176^T.

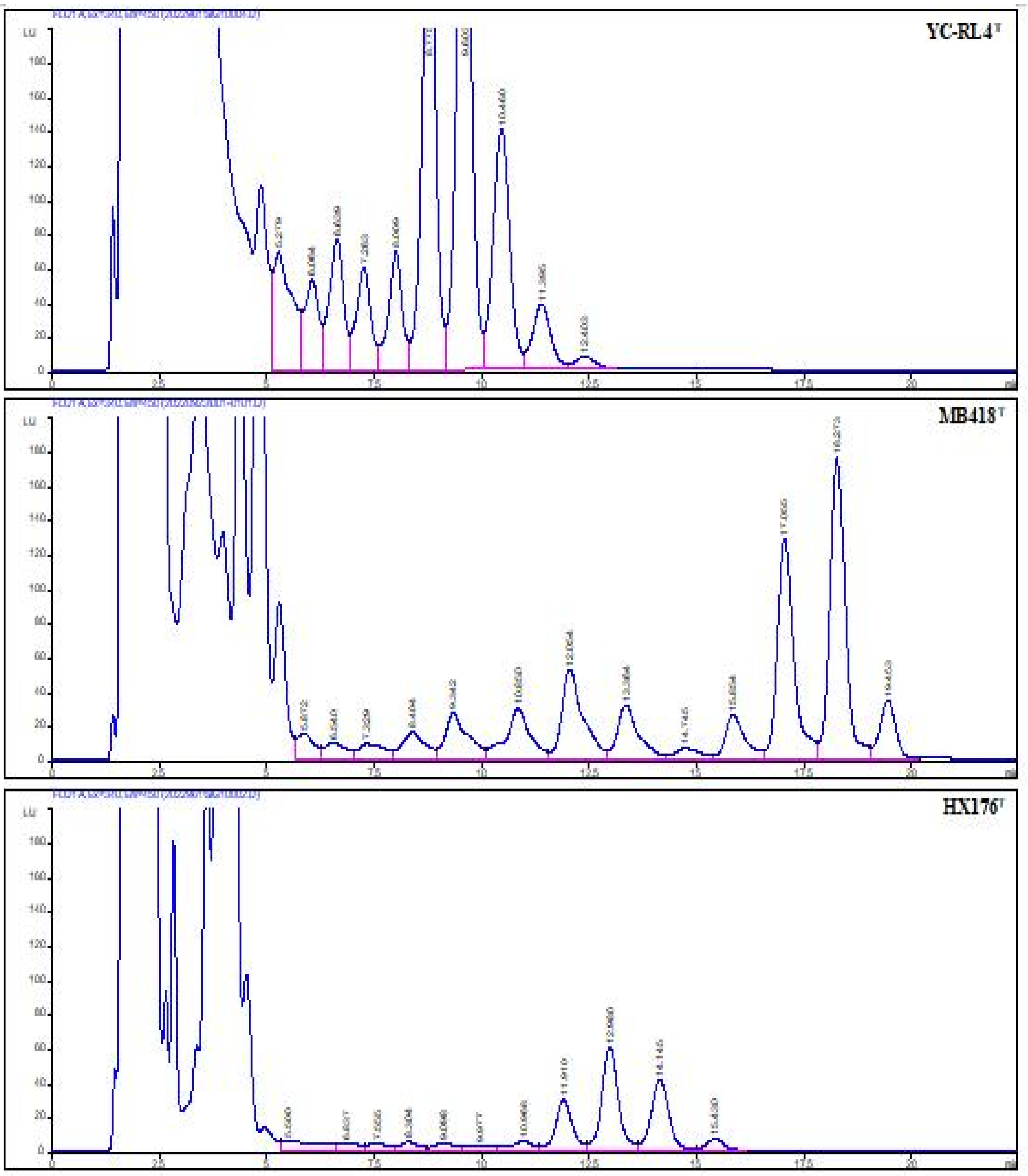

Supplementary Figure S3. Maximum-Likelihood phylogenomic tree showing the phylogenetic positions of strains YC-RL4^{T}, MB418^{T} and HX176^{T} within the *Mycobacterium* genus based on conserved core genes.

Bootstrap values are shown at branch nodes as a percentage of 1,000 bootstrap replicates. RefSeq assembly accession numbers for *Mycobacterium* references are shown in brackets. *Streptomyces albus* CAS922^{T} (RefSeq assembly accession no. GCF 011006355.1) was used as the outgroup (not shown in the figure). Bar indicates 0.07 substitutions per nucleotide position.

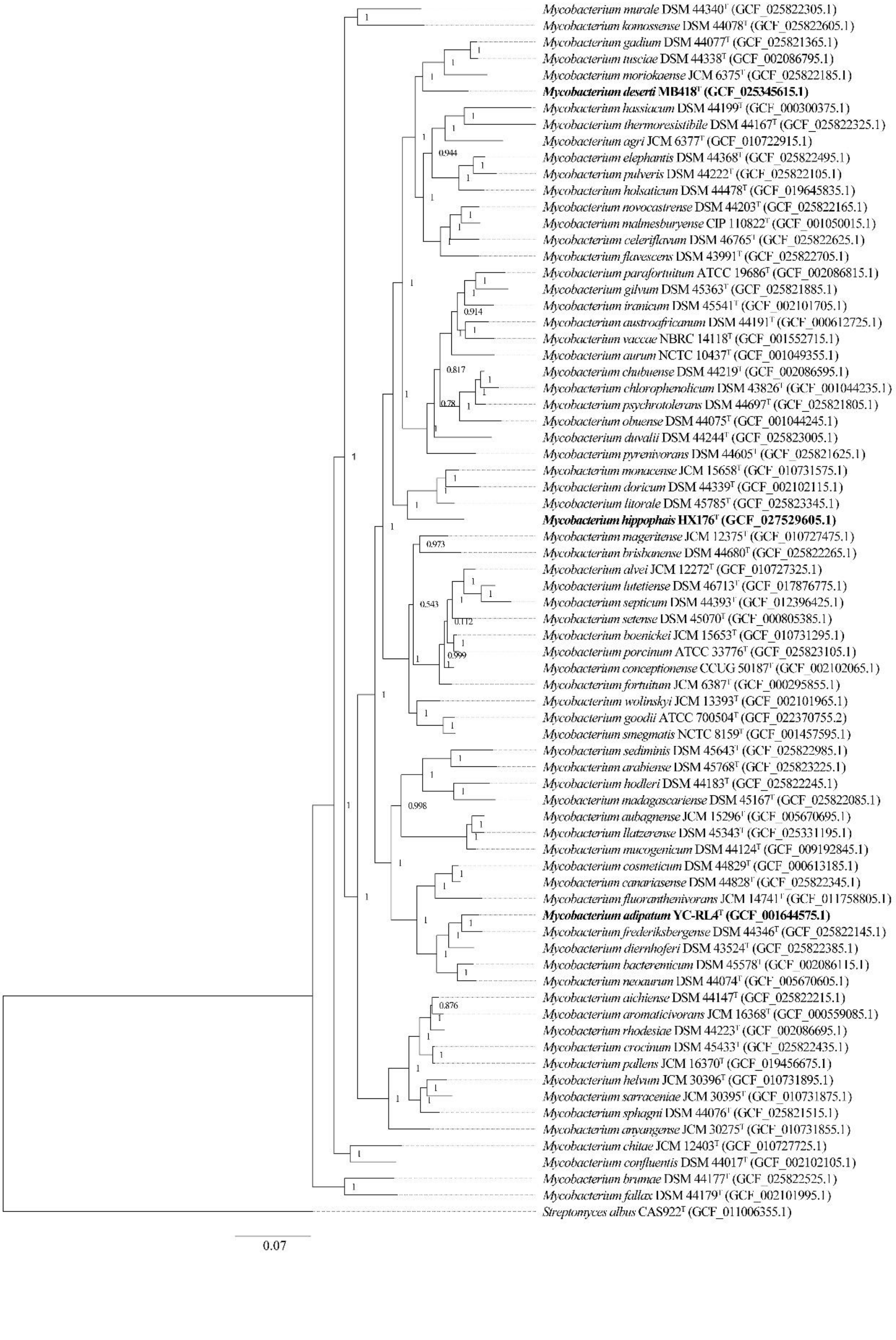

Supplementary Table S1. Cellular fatty acid profiles of strains YC-RL4^T, MB418^T, and HX176^T and other phylogenetically-related strains.

Numbers indicate percentages of total profiles for each component in each strain. Strain designations are: 1, YC-RL4^T; 2, *M. fluoranthenivorans* JCM 14741^T; 3. *M. frederiksbergense* DSM 44346^T; 4, MB418^T; 5, HX176^T; and 6, *M. litorale* DSM 45785^T.

All data were generated in this study except for that indicated by "a" that is from Hormisch et al., 2004.

-, not detected.

**Fatty acid**	1	2[a]	3	4	5	6
**Saturated**						
$C_{14:0}$	8.2	10.0	7.3	-	-	2.9
$C_{16:0}$	15.3	42.1	11.2	1.9	8.8	20.3
$C_{18:0}$	-	3.4		-	-	-
$C_{20:0}$	-	1.1	1.1	-	-	-
**Unsaturated**						
$C_{16:1}$ B	1.7	-	2.4	-	-	-
$C_{16:1}\omega 6c$	-	4.6	-	-	-	-
$C_{16:1}\omega 7c$	-	13.5	-	-	-	1.5
$C_{17:1}\omega 7c$	15.3	-	46.8	47.7	43.2	19.1
$C_{18:1}\omega 9c$	7.9	16.8	18.4	2.8	3.9	11.8
$C_{16:0}$ 10-methyl	-	2.2	-	-	-	-
$C_{18:0}$ 10-methyl	5.8	5.1	-	2.9	3.4	6.8
$C_{19:1}$ trans 7	-	-	-	-	30.9	-
**Others**						
Sum in feature 4 (anteiso-$C_{17:1}$ B/iso-$C_{17:1}$ I)	8.3		8.0	1.5	6.6	13.7
Sum in feature 5 (anteiso-$C_{17:1}$ B/iso-$C_{17:0}$)	-	-	1.1	-	3.3	2.2
Sum in feature 7 ($C_{19:1}\omega 6c$/unknown equivalent chain length [ECL] 18.846)	-	-	-	-	-	1.8
Sum in feature 9 (iso- $C_{17:1}\omega 9c$/$C_{16:0}$ 10-methyl)	3.2	-	2.7	43.3	-	15.2

Supplementary Table S2. 16S rRNA gene nucleotide sequence similarities (%) shared between genes of strains YC-RL4^{T}, MB418^{T}, HX176^{T}, and phylogenetically-related *Mycobacterium* strains.

Species designations are as follows: 1, YC-RL4^{T}; 2, MB418^{T}; 3, HX176^{T}; 4. *M. fluoranthenivorans* JCM 14741^{T}; 5, *M. frederiksbergense* DSM 44346^{T}; 6, *M. canariasense* DSM 44828^{T}; 7, *M. diernhoferi* DSM 43524^{T}; 8, *M. bacteremicum* DSM 45578^{T}; 9, *M. neoaurum* DSM 44074^{T}; 10, *M. celeriflavum* DSM 46765^{T}; 11, *M. moriokaense* JCM 6375^{T}; 12, *M. tusciae* DSM 44338^{T}; 13, *M. litorale* DSM 45785^{T}; 14, *M. doricum* DSM 44339^{T}; and 15, *M. monacense* JCM 15658^{T}.

16S rRNA gene nucleotide sequence similarities (%) > 98.65% are highlighted in bold.

Species	1	2	3	4	5	6	7	8	9	10	11	12	13	14	15
1	**100.0**														
2	97.2	**100.0**													
3	98.0	98.6	**100.0**												
4	**99.3**	97.0	96.6	**100.0**											
5	**99.2**	97.2	96.8	**98.8**	**100.0**										
6	98.6	97.0	96.7	**99.0**	**98.8**	**100.0**									
7	98.5	96.2	95.8	98.5	98.5	**98.8**	**100.0**								
8	**98.8**	97.4	96.9	98.6	**98.7**	**99.0**	98.5	**100.0**							
9	**99.1**	97.3	96.7	**98.8**	98.6	98.7	98.3	**99.7**	**100.0**						
10	97.4	98.5	97.6	97.2	97.5	97.4	96.6	98.0	97.8	**100.0**					
11	97.6	98.5	97.9	97.3	97.8	97.6	96.8	97.9	97.7	**99.3**	**100.0**				
12	97.2	97.9	96.7	97.0	97.0	96.9	96.5	97.9	98.0	96.9	97.2	**100.0**			
13	97.2	98.3	98.3	97.0	96.7	96.6	96.0	97.1	97.1	97.4	97.3	97.4	**100.0**		
14	96.5	97.4	97.5	96.4	96.1	96.3	95.6	96.5	96.4	96.9	97.2	96.7	98.2	**100.0**	
15	96.8	97.9	97.7	96.5	96.6	96.8	96.2	97.0	97.0	97.2	97.7	97.3	98.5	**99.2**	**100.0**

Supplementary Table S3. Genomic features of strains YC-RL4^T, MB418^T, HX176^T, and phylogenetically-related *Mycobacterium* strains.

Strain designations are as follows: 1, YC-RL4^T; 2, *M. fluoranthenivorans* JCM 14741^T; 3. *M. frederiksbergense* DSM 44346^T; 4, MB418^T; 5, HX176^T; and 6, *M. litorale* DSM 45785^T. *: Data are derived from this study.

	1	2	3	4	5	6
Isolation source	Petroleum -contaminated soil	Contaminated soil of a former coal gas plant	Coal tar-contaminated soil	Gravel soil	Rhizosphere soil	Soil
No. of contigs	2	6	96	15	41	13
N50 Length (bp)	5,801,417	3,695,880	195,370	710,882	302,245	4,232,783
Genome size (Mbp)	6.1	6.4	6.4	5.6	5.9	5.6
No. of CDS	5,634	5,888	5,944	5,444	5,575	5,234
G+C (%)	67.4	67.0	67.1	66.5	69.3	68.9
DDBJ/EMBL/GenBank accession number of draft genome	CP015596.1	JAANOW000000000.1	JACKTH000000000.1	JAODWD000000000.1	JAPZPY000000000.1	JACKVO000000000.1
GenBank/RefSeq assembly accession number	GCA_001644575.1	GCA_011758805.1	GCA_025822145.1	GCA_025345615.1	GCA_027529605.1	GCA_025823345.1

Supplementary Table S4. Average nucleotide identity (ANI) and digital DNA–DNA hybridization (dDDH) (%) values between strains YC-RL4^T, MB418^T, HX176^T and related strains of the genus *Mycobacterium*.

1, YC-RL4^T; 2, MB418^T; 3, HX176^T; 4. *M. fluoranthenivorans* JCM 14741^T; 5, *M. frederiksbergense* DSM 44346^T; 6, *M. canariasense* DSM 44828^T; 7, *M. diernhoferi* DSM 43524^T; 8, *M. bacteremicum* DSM 45578^T; 9, *M. neoaurum* DSM 44074^T; 10, *M. celeriflavum* DSM 46765^T; 11, *M. moriokaense* JCM 6375^T; 12, *M. tusciae* DSM 44338^T; 13, *M. litorale* DSM 45785^T; 14, *M. doricum* DSM 44339^T; 15, *M. monacense* JCM 15658^T. ANI values > 95% and dDDH values > 70% are highlighted in bold.

Strain	ANI (%)			dDDH (%)		
	1	2	3	1	2	3
1	**100.0**			**100.0**		
2	76.1	**100.0**		19.4	**100.0**	
3	77.4	78.4	**100.0**	20.5	21.1	**100.0**
4	79.5	76.0	77.3	22.4	19.7	20.5
5	86.9	76.2	77.6	31.8	19.6	20.3
6	78.0	76.6	77.7	23.0	19.9	20.9
7	83.2	76.2	77.7	26.2	19.7	20.7
8	81.8	76.3	77.4	24.5	19.7	20.5
9	80.7	75.6	76.8	23.3	19.4	20.1
10	77.0	80.0	78.5	20.4	22.6	21.1
11	76.2	79.4	78.0	19.7	22.0	20.8
12	76.0	79.0	77.6	19.6	21.6	20.6
13	77.3	78.3	80.1	20.4	20.9	22.7
14	77.1	77.7	80.0	20.9	21.0	22.4
15	77.3	78.0	80.0	20.7	20.8	22.6

Supplementary Table S5. Genetic features predicted in the genomes of strains YC-RL4^T, MB418^T, and HX176^T that may be involved in PAH degradation and heavy metal resistance.

Gene	Protein	YC-RL4^T	MB418^T	HX176^T
**PAH degradation**				
	Aromatic ring-hydroxylating dioxygenase subunit alpha	A7U43_RS23410, A7U43_RS03875, A7U43_RS03905, A7U43_RS11250	N4S67_16880, N4S67_19850, N4S67_27040, N4S67_27205, N4S67_27385, N4S67_27920, N4S67_03555	O6P37_RS03225, O6P37_RS07785, O6P37_RS07815, O6P37_RS07845, O6P37_RS07995, O6P37_RS11275, O6P37_RS18080, O6P37_RS18635
phtAa	Phthalate 3,4-dioxygenase subunit alpha	A7U43_RS01735	N4S67_06110	-
pcaC	4-carboxymuconolactone decarboxylase	A7U43_RS27830, A7U43_RS01860	N4S67_17815	O6P37_RS10995
pcaD	3-oxoadipate enol-lactonase	-	N4S67_26015	-
pcaG	Protocatechuate 3,4-dioxygenase subunit alpha	A7U43_RS27840, A7U43_RS01870	N4S67_17825	O6P37_RS11005
pcaH	Protocatechuate 3,4-dioxygenase subunit beta	A7U43_RS27845, A7U43_RS01875	N4S67_17830	O6P37_RS11010
**Heavy metal resistance**				
copC	Copper resistance protein	A7U43_RS03135, A7U43_RS04605	N4S67_24355	O6P37_RS20935
copD	Copper resistance protein	-	N4S67_24350	O6P37_RS20940
merA	Mercury(II) reductase	A7U43_RS01925	-	-
merB	Organomercurial lyase	A7U43_RS01935	-	-

Supplementary Table S6. Biosynthetic gene clusters on contigs longer than 10 kbp in the genomes of strains YC-RL4^T, MB418^T, and HX176^T.

Genomic location	Type	From (bp)	To (bp)	Secondary metabolite synthesis gene cluster	Nucleotide similarity
**YC-RL4^T**					
NZ_CP015596.1_1 Region 1.1	T1PKS	192,228	236,895	Glycopeptidolipid	26%
NZ_CP015596.1_Region 1.2	Terpene	757,718	776,745	/	/
NZ_CP015596.1_Region 1.3	NRPS-like	924,872	967,120	/	/
NZ_CP015596.1_Region 1.4	T1PKS	1,016,726	1,060,687	/	/
NZ_CP015596.1_Region 1.5	NAPAA	1,312,005	1,345,889	ε-Poly-L-lysine	100%
NZ_CP015596.1_Region 1.6	RiPP-like	1,586,528	1,597,325	TVA-YJ-2	4%
NZ_CP015596.1_Region 1.7	T1PKS,NRP-metallophore,NRPS	2,637,363	2,704,031	Mycobactin	80%
NZ_CP015596.1_Region 1.8	T3PKS	3,071,631	3,111,498	Alkylresorcinol	66%
NZ_CP015596.1_Region 1.9	Ectoine	3,516,229	3,526,618	Ectoine	100%
NZ_CP015596.1_Region 1.10	T1PKS	3,629,688	3,675,891	Kendomycin B	6%
NZ_CP015596.1_Region 1.11	NRPS-like, NAGGN	4,173,826	4,214,307	/	/
NZ_CP015596.1_Region 1.12	NRPS-like	4,976,776	5,019,421	Tetrocarcin A	6%
NZ_CP015596.1_Region 1.13	Terpene	5,157,675	5,178,619	Isorenieratene	71%
NZ_CP015596.1_Region 1.14	Redox-cofactor	5,303,872	5,326,587	Salinomycin	6%
NZ_CP015596.1_Region 1.15	T3PKS	5,644,695	5,685,783	Alkylresorcinol	100%
**MB418^T**					
Scaffold1 Region 1.1	T1PKS	565,360	611,629	Glycopeptidolipid	20%
Scaffold1 Region 1.2	NAPAA	1,185,659	1,210,185	ε-Poly-L-lysine	100%

Scaffold2 2.1	Region	Redox-cofactor	206,909	229,603	Salinomycin	6%
Scaffold2 2.2	Region	T1PKS	845,505	896,556	Glycopeptidolipid	23%
Scaffold3 3.1	Region	NRPS-like	287,203	328,520	/	/
Scaffold3 3.2	Region	NRPS-like	595,098	637,295	/	/
Scaffold3 3.3	Region	PKS-like	690,975	732,024	Diastaphenazine/izumiphenazine C	10%
Scaffold3 3.4	Region	NRPS	795,742	845,230	Terpenibactin A/terpenibactin B/terpenibactin C	71%
Scaffold4 4.1	Region	NRPS	1	28,781	/	/
Scaffold4 4.2	Region	NRP-metallophore,NRPS,T1PKS	395,623	453,331	Mycobactin	50%
Scaffold5 5.1	Region	Terpene	187,418	208,356	Isorenieratene	71%
Scaffold6 6.1	Region	T1PKS	335,654	381,113	/	/
Scaffold7 7.1	Region	NRPS	1	22,844	Depsibosamycin B/depsibosamycin C/depsibosamycin D	9%
Scaffold10 10.1	Region	NRPS	1	1,944	/	/
**HX176^T**						
Scaffold1 1.1	Region	RiPP-like	803,873	814,670	TVA-YJ-2	4%
Scaffold1 1.2	Region	NAPAA	1,043,167	1,077,081	ε-Poly-L-lysine	100%
Scaffold1 1.3	Region	NRPS	1,134,177	1,161,583	Oryzanaphthopyran A/oryzanaphthopyran B/oryzanaphthopyran C/oryzanthrone A/oryzanthrone B/chlororyzanthrone A/chlororyzanthrone B	6%
Scaffold2	Region	NRPS	90,344	154,318	Glycopeptidolipid	10%

2.1						
Scaffold2 2.2	Region	NRP-metallophore,NRPS,T1PKS	161,785	229,075	Mycobactin	80%
Scaffold3 3.1	Region	Redox-cofactor	25,730	48,461	Salinomycin	6%
Scaffold3 3.2	Region	Mycosporine-like	202,248	245,596	Shinorine/4-deoxygadusol/mycosporine glycine	75%
Scaffold3 3.3	Region	T1PKS	451,877	498,281	Lobosamide A/lobosamide B/lobosamide C	8%
Scaffold4 4.1	Region	T3PKS	37,506	78,600	Lagunapyrone A/lagunapyrone B/lagunapyrone C	22%
Scaffold4 4.2	Region	T1PKS	138,347	184,637	Calicheamicin	2%
Scaffold5 5.1	Region	T1PKS	81,253	132,373	Glycopeptidolipid	26%
Scaffold6 6.1	Region	NRPS,T1PKS	1	130,372	Glycopeptidolipid	20%
Scaffold11 11.1	Region	Terpene	26,629	47,612	Isorenieratene	71%
Scaffold12 12.1	Region	NRPS-like	1	30,063	/	/
Scaffold13 13.1	Region	NRPS	1	25,703	Nocardiopsistin A/nocardiopsistin B/nocardiopsistin C	9%
Scaffold13 13.2	Region	T1PKS	53,168	96,247	4-hexadecanoyl-3-hydroxy-2-(hydroxymethyl)-2H-furan-5-one	18%
Scaffold13 13.3	Region	T1PKS	112,971	158,436	/	/
Scaffold14 14.1	Region	NRPS-like	30,896	73,550	/	/
Scaffold20 20.1	Region	T1PKS	13,841	41,869	Glycopeptidolipid	10%
Scaffold22 22.1	Region	NRPS	1	27,176	Glycopeptidolipid	20%
Scaffold25 25.1	Region	NRPS	1	3,793	/	/
